# Community acceptability of cardiovascular risk screening in faith centres in the Kassena-Nankana districts of Northern Ghana: a qualitative study

**DOI:** 10.1186/s12889-025-24780-z

**Published:** 2025-11-05

**Authors:** Samuel Tamti Chatio, Natalie Darko, Sawudatu Zakariah- Akoto, Andy Willis, Engelbert A Nonterah, Ceri R Jones, Joseph Alale Aweeya, Ffion Curtis, Setor Kunutsor, Samuel Seidu, Patrick O Ansah

**Affiliations:** 1 Navrongo Health Research Centre, Ghana Health Service, Navrongo, Ghana; 2 School of Medical Sciences, Division of Global, Lifestyle and Metabolic Health, Leicester Diabetes Centre, University of Leicester, Leicester, UK; 3 Nutrition Department, Noguchi Memorial Institute for Medical Research, College of Health Sciences, University of Ghana, Accra, Ghana; 4 School of Public Health & HRB Clinical Research Facility, University College Cork, Cork, Ireland; 5 Liverpool Reviews & Implementation Group (LRiG), Institute of Population Health, University of Liverpool, Liverpool, UK; 6 Department of Internal Medicine, Section of Cardiology, Rady Faculty of Health Sciences, Max Rady College of Medicine, University of Manitoba, Saint Boniface Hospital, Winnipeg, Manitoba, Canada; 7 Faculty of Business and Law, Birkbeck University of London, Bloomsbury, London, UK; 8 Julius Global Health, Julius Centre for Health Sciences and Primary Care, University Medical Centre Utrecht, Utrecht University, Utrecht, Netherlands; 9 Department of Epidemiology, School of Public Health, CK Tedam University of Technology and Applied Sciences, Navrongo, Ghana

**Keywords:** Faith-based screening, Type 2 diabetes, Cardiovascular risk factors, Community acceptability, Northern Ghana

## Abstract

**Purpose:**

This study explored the community acceptability of a faith-based screening programme for cardiometabolic risk factors, specifically type 2 diabetes and hypertension, among faith congregation members in Northern Ghana.

**Methods:**

The research team conducted 18 in-depth semi-structured qualitative interviews and 10 focus groups, with a total of 123 participants, between October 2022 and February 2023 to discuss the acceptability of a proposed faith-based screening programme. Participants included faith leaders, congregation member nurses, and other congregation members from six religious organizations in the Kassena-Nankana East Municipality and West District in the Upper East Region of Northern Ghana. The study population ranged from 18 to 85 years old. The analytical process involved a combined inductive and deductive thematic analysis to identify key themes related to community acceptability of cardiovascular risk screening.

**Results:**

Participants’ comments on the acceptability of the proposed screening programme centred on four key themes: (1) Awareness and perception of type 2 diabetes and hypertension, (2) Perceptions of current screening services, (3) Challenges in Implementation, (4) Implementation strategies (what might work). All participants expressed a willingness to participate in the programme, though its success was deemed contingent on the provision of free screening services, access to treatment, and addressing certain challenges: stigma surrounding diagnosis, and concerns about treatment affordability. Solutions included support from congregation health professionals in delivering screenings, access to diabetes treatment, and assurances of maintaining patient confidentiality.

**Conclusions:**

The findings highlight community acceptability and willingness to participate in the screening programme, contingent upon addressing key challenges identified through both focus group discussions and in-depth interviews. These challenges included concerns about treatment affordability, the need for comprehensive community education, maintaining confidentiality, and ensuring screening services are convenient and time efficient. Participants emphasized the importance of leveraging congregation-based health professionals, providing pre- and post-screening counselling, and offering follow-up support for those diagnosed. These insights demonstrate that while faith-based centres present a promising platform for health interventions. The findings highlight the need for multi-sectoral collaboration to ensure equitable access, referral, and follow-up across health and community systems, addressing these barriers is crucial for successful implementation and sustainability.

**Supplementary Information:**

The online version contains supplementary material available at 10.1186/s12889-025-24780-z.

## Introduction

The global burden of non-communicable diseases (NCDs) has increased to an unprecedented scale and poses both major health and socioeconomic threats. An estimated 1.5 million deaths were caused directly by diabetes each year, with 80% of deaths occurring in low- and middle-income countries over the past few decades [1]. Asia and Africa have been identified as regions with the greatest potential increases in mortality, where the rates could rise to 2 or 3 times that of the present day. Sub-Saharan Africa is projected to experience the steepest rise in type 2 diabetes prevalence, with estimates suggesting a 129% increase by the year 2045 [2]. This alarming trend is expected to worsen the already significant prevalence of complications and comorbidities associated with type 2 diabetes in the region [3]. Ghana is experiencing a double burden of disease characterised by persistent communicable diseases and more recently, rising levels of NCDs such as obesity, type 2 diabetes, and hypertension [4–6]. Northern Ghana has seen a rise in the prevalence of these conditions. Within the Navrongo Health and Demographic Surveillance Systems’ coverage area, the prevalence of diabetes among middle-aged men and women is about 4.8% with observed high level of lack of awareness, low treatment, and poor control [7]. One approach to increasing diabetes and hypertension awareness and consequently apply prevention strategies, is the implementation of a cardiovascular screening programme through faith-based centres, which are deeply ingrained in the social and cultural fabric of these communities.

Faith-based institutions, including churches, mosques, and traditional healing centres, have a significant influence and reach within communities, positioning them as pivotal platforms for health promotion and disease prevention. These centres provide a trusted and familiar environment, making them conducive for initiatives like cardiovascular screenings. Historically, many of these institutions have emphasized health, evident from their support groups and health ministries [8]. In Northern Ghana, these organizations are prominent and play a major role in shaping health behaviours, making them well-suited for disseminating health information, conducting screenings, and delivering educational programmes [9]. Faith-based institutions are typically characterized by their expression of religious creeds, shared values, worship activities, and community-driven initiatives aimed at improving social well-being [10]. Their commitment to holistic care—addressing physical, spiritual, and social needs—further enhances their role as credible platforms for health interventions. Past studies have highlighted the efficacy of faith-placed approaches: intertwining religious activities with disease risk education and the deployment of faith-based interventions, both within and outside religious settings [11]. For example, a faith-based lifestyle programme in South Africa received community approval and showed potential for weight loss [12], while other studies on culturally tailored education in Black communities, highlight benefits like improved health literacy and weight loss through integrating religious practices with community resources [13, 14].

However, community acceptability, which encompasses factors such as community knowledge, support, available resources, community assets and attitudes are critical factors in the successful implementation of health programmes [15–18]. In the context of implementing a screening programme in the Kasena Nankana Districts, it is important to understand and enhance community acceptability to ensure the programme’s effectiveness and sustainability. By addressing the community’s concerns, fostering trust, and actively involving community members in the planning and decision-making processes, the programme can gain acceptance and participation from the community, leading to its success. While previous studies have explored faith-based health interventions, there is limited evidence on how community acceptability is shaped in the early stages of designing cardiovascular screening programmes in Northern Ghana. This study addresses this gap by focusing on a predominantly rural, multi-faith setting, and by capturing perspectives across multiple stakeholder groups—including religious leaders, healthcare workers, and community members. This study therefore aimed to assess the acceptability of a proposed faith-based cardiovascular disease screening model in Northern Ghana.

## Method

This study was part of a broader project aimed at piloting the feasibility of screening for hypertension and Type 2 Diabetes Mellitus (T2DM) within faith centres and facilitating referrals to the health system in the Kassena-Nankana districts of Northern Ghana [17, 18]. This broader project was comprised of four phases, with the first phase being a community asset mapping exercise to identify resources that would support the faith-based model of education, screening, and referral. The second phase; being the current study which aims to; assess the community acceptability of the proposed faith-based and/or faith-placed screening and prevention pathway. Following on from the current study, phase three will involve the delivery of educational support to religious leaders, congregation member nurses, and peer leaders, to facilitate delivering of medico-religious messaging to faith communities. Phase four will involve the implementation of type 2 diabetes and cardiovascular risk factor screening services in faith centres to identify those at high risk.

The current study focuses on the qualitative assessment of community acceptability of the proposed faith-based and/or faith-placed screening and prevention pathway. To provide contextual insights that inform community acceptability of screening, we also explored participants’ awareness and perceptions of diabetes and its prevention and management. Insights gathered are essential for understanding the community’s readiness and practical considerations for implementation.

### Study design

A qualitative approach was used, involving semi-structured interviews and focus group discussions (FGDs) with faith leaders, congregation member nurses, and congregation members from six religious organizations. Braun and Clarke’s [23] reflexive thematic analysis approach was employed, discussed further below. This method allowed for an in-depth understanding of the community’s perspectives on the acceptability of the proposed screening services for type 2 diabetes and hypertension at selected religious organizations in the area, where such services had not previously existed.

#### Study setting

This study was conducted in the Kassena-Nankana East Municipality (KNEM) and Kassena-Nankana West District (KNWD) in the Upper East Region of Ghana. The two districts cover an area of 1,675 square kilometres, with a population of approximately 162,000 people under surveillance by the Navrongo Health and Demographic Surveillance System (NHDSS). The NHDSS was established in 1992 by the Navrongo Health Research Centre (NHRC), one of three research centres under the Ghana Health Service. It monitors health and demographic dynamics in these districts, facilitating the evaluation of health and social interventions. The NHDSS systematically collects longitudinal data to track disease burden, health service utilization, and mortality trends, making it a key resource for research and public health planning in northern Ghana [19].

The population in these districts is predominantly rural, with subsistence farming as the main economic activity. Several religious organizations operate in the area, with Christianity being the dominant faith. According to the 2021 Population and Housing Census and current unpublished data from the Navrongo Health and Demographic Surveillance System, about 71% of the population is Christian, 20% Muslims, 3% traditional religion and 6% belong to other religions. (ref) [20, 21]. Faith-based organizations play an essential role in social engagement, health promotion, and community support, making them suitable platforms for implementing health interventions.

Ghana’s healthcare system is structured into four tiers: Community-based Health Planning and Services (CHPS) compounds, health centres, district hospitals, and tertiary hospitals. The National Health Insurance Scheme (NHIS) was introduced to improve healthcare affordability and reduce financial barriers. However, many people, particularly in rural areas, remain uninsured, requiring out-of-pocket payments for consultations, diagnostic tests, medications, and specialist services. Even for NHIS subscribers, some essential health services and medications are not fully covered, which can limit access to comprehensive care.

#### Sampling and recruitment

Faith leaders, congregation member nurses, church elders, youth leaders and congregation members aged 18 −75years were recruited to the study from October 2022 to February 2023. Congregation members took part in the focus groups (1–2 h) while the interviews (1–1.5 h) were conducted with faith leaders, congregation members nurses, church elders and youth leaders. A purposive sampling method was used to select six faith-based centres and participants for the interviews. Additionally, the programme sought to identify mechanisms and resources for programme delivery and scalability at the selected faith-based centres. Therefore, six faith-based centres were selected based on the population of each of these religious organizations. Information on membership strength of faith-based centres was obtained from mapping exercise conducted prior to the implementation of the study. The selection of these organizations was also driven by the need to capture diverse perspectives and ensure representation across denominations, which is critical to understanding the broader community acceptability of the screening programme. In the two districts, several religious groups/denominations were identified during the mapping exercise. This included those of Muslim faith and Christian faith such as Roman Catholics and protestants denominations (e.g. Pentecostals, Presbyterians and Jehovah Witnesses). From those that were eligible, we purposively selected three (3) religious organizations of varying denominations, in each district -giving a total of six (6) for the study. However, the majority of the faith-based centres in the two districts are Christians.

Two research assistants from the local communities, were recruited and trained on the rationale of the study, data collection and culturally sensitive informed consent procedures, including translating interview guides into the two main local languages (Kasem and Nankani) spoken in the study area. This was done to facilitate consistency during data collection. The training was conducted by senior members of the research team, including experienced qualitative researchers (ND, SZ, AW and SC), who provided comprehensive guidance on interview techniques, ethical considerations, and qualitative research methods. These senior researchers also actively supported the research assistants throughout data collection to ensure methodological rigor. A pilot interview was conducted at the end of the training with a community faith member, who collaborated with the study team to refine and finalize the interview guide for data collection. The questions were collaboratively produced and informed by the community member, ensuring they were appropriate and considerate of potential low levels of literacy and health literacy in the study population. (Please see supplementary files for all the interview guides).

The recruitment process was facilitated by a local community engagement officer who was trusted by the local communities and had established collaborations and partnerships with community members and organizations. This approach was guided by the *NIHR Guiding Principles for Community Engagement and Involvement in Global Health Research* [22].

#### Data collection

Prior to data collection, the study team conducted visits to all the selected faith-based centres in the two districts to inform faith leaders about the study and obtain their permission and support to conduct the interviews. The research team then visited the selected religious organizations and introduced themselves to faith leaders and some congregation members before the interviews were conducted. Interviews and focus groups with faith leaders, congregation member nurses, and congregation members were coordinated by the community engagement officer and conducted by the research assistants, and experienced qualitative team members (SC, ND, AW and AZ) at faith-based centres after obtaining informed consent. Written and verbal consent were completed, and interviews were audio recorded with participants’ permission. Research assistants, and experienced qualitative team members, ensured accurate data collection throughout the process. A total of 10 focus groups and 18 interviews were conducted with participants. Further information regarding category and number of interviews conducted is summarised in Table 1 and demographic characteristics of participants is provided in Table 2. Data collection continued until data saturation was reached, determined by the point at which no new themes, perspectives, or variations in responses emerged from additional interviews and focus groups. The research team continuously assessed saturation through concurrent data analysis and discussions.

**Table 1:** Category and number of focus groups and interviews conducted

Type of interview	Category	No.of IDIs and FDGs
Interviews	Faith leaders	6
	Congregation member Nurses	6
	Church Elders	3
	Youth and women leaders	3
Total		**18** (18 participants)
Focus Groups	Male congregation members	5
	Female congregation members	5
Total		**10** (105 participants)

Participants were predominantly from the 29–39 (36.6%) and 40–50 (28.5%) years age groups and the majority were married (67.5%). Participants were equally distributed across education categories and the majority were employed in farming (30.8%) or trader (30.5%) industry. Participants were predominantly of Christian faith (77.2%), with muslim participants in the minority (22.8%). The majority of participants were of Kasem (72.4%) or Kassena (10.6) ethnicity, with several other minority ethnic groups also represented. Demographic figures for religious and ethnic background are similar to the population as a whole, in the study locality.

**Table 2:** Demographic characteristics of participants

Variable	Frequency *n* (%)
Sex	
Male	60 (48.8)
Female	63 (51.2)
Age Group	
18–28	11 (8.9)
29–39	45 (36.6)
40–50	35 (28.5)
51–61	23 (18)
62–75	9 (7)
Marital status	
Married	**86 (67.5)**
Single	21 (17.1)
Divorced	4 (3.3)
Separated	1 (0.8)
Widowed	9 (7.3)
Level of Education	
Never been to school	22 (17.9)
Primary	32 (25.2)
Secondary/Technical	38 (30.1)
Tertiary/Professional	31 (25.2)
Occupation	
Unemployed Civil/public servant Farmer/artisan Trader	12(9.8) 30 (24.4) 38 (30.8) 43 (35.0)
Religion	
Muslim	28 (22.8)
Christian	95 (77.2)
Ethnicity	
Kasem	89 (72.4)
Bulisa	2 (1.6)
Nankam	2 (1.6)
Kusase	1 (0.8)
Gurune	1 (0.8)
Dagati	1 (0.8)
Kassena	13 (10.6)
Akan	5 (4.1)
Ewe	1 (0.8)
Mamprusi	5 (4.1)
Mose	2 (1.6)
Buli	1 (0.8)

The interview guide for the FGDs and IDIs with religious leaders and congregation members contained open-ended questions addressing several key areas: perceptions and knowledge of diabetes, its prevention and management, the role of screening programmes in detecting diabetes and other cardiovascular risk factors, and the support services required for effective screening. The interview guides were developed based on the objectives of the study. The guides were developed by the first, second and fourth authors and validated by the other co-authors. Table 3 shows a sample of the questions addressing perceptions and acceptability of screening. (Please see supplementary files for the interview guides, and sample transcript for FGD 1, female congregation members).

**Table 3:** Interview guide questions on perceptions of the use of faith-based centres for diabetes screening

1. What do you think about us screening for diabetes using faith centres such as Churches and Mosques in this district?
2. Which people do you think could be used for the screening exercise? Probe for the use of faith centres health professionals e.g. If it is possible to screen people for diabetes through the church, then which people should be used for the screening exercise? Should it be faith-based health professionals like the nurses in the church…
3. What do you think will be the challenges in implementing this programme?
4. What concerns do you think people will have knowing their diabetes status?
5. How would you want the information on the diabetes status of people to be shared with them?
6. What factors do you think are likely to affect acceptance of the screening exercise?
7. What suggestions would you recommend to improve acceptability of the exercise/make this screening exercise more acceptable?

### Ethics approval

 Ethical approval was granted by Navrongo Health Research Centre Institutional Ethics Review Board Ref: NHRCIRB47I on 27/07/22. Prior to participation in an interview or focus group discussion, informed consent was collected from all participants.

### Data management and analysis procedures

This study followed Braun and Clarke’s [23] six-step framework for reflexive thematic analysis to guide the analytical process, including familiarisation, generating initial codes, searching for themes, reviewing themes, defining and naming themes, and producing the analysis report. All interviews were audio-recorded and transcribed verbatim. Transcriptions were conducted by research assistants who were fluent in both English and the local languages. Interviews and focus groups were conducted in English or the local languages, depending on participants’ language preference. For non-English interviews, the research team and bilingual research assistants translated and transcribed the data to ensure accuracy and consistency. To maintain translation accuracy, a back translation process was conducted, where translated interview guides were independently back translated into English and checked by a senior bilingual researcher (KT). Discrepancies were discussed and resolved to ensure conceptual equivalence and linguistic accuracy. A codebook containing main and sub-themes was developed using a combined inductive and deductive approach to facilitate data coding and thematic analysis with QSR NVivo 12 software. The codebook was developed using a combination of established categories based on the original research objectives and emergent themes from the data. Deductive coding was guided by the study objectives and interview questions, while additional themes emerged inductively from participants’ responses. Two researchers independently coded all transcripts, followed by discussions to resolve discrepancies and ensure thematic coherence. To ensure a rigorous and trustworthy interpretation of the data, an inter-coder agreement process was undertaken, and a comparison of identified themes was conducted to enhance reliability. Discrepancies were resolved through discussion, ensuring consistency in coding and theme development.

To further ensure trustworthiness, we adhered to the four criteria of qualitative rigor: credibility, dependability, confirmability, and transferability. This was achieved through multiple strategies, including maintaining an audit trail of coding decisions, conducting peer debriefing, and engaging in reflexive discussions throughout the analysis process. Member-checking was also conducted with selected participants to enhance the credibility of findings. Member-checking was conducted with selected participants by sharing preliminary themes to verify the accuracy and resonance of interpretations, rather than full transcript review. The major and sub-themes are presented as a narrative, supported by illustrative quotes from the data.

## Results

Data were coded into four overarching themes: awareness and perceptions of Type 2 diabetes and hypertension; perceptions of screening services; challenges in implementation; and addressing implementation challenges. Each theme is presented below with illustrative quotes from focus groups and interviews. A summary of the themes and subthemes is presented in Table 4.

**Table 4:** Summary of themes and subthemes

Theme	Subthemes
1. Awareness and Perceptions of Type 2 Diabetes and Hypertension	• Misconceptions about causes and risk • Limited understanding of prevention • Fear and uncertainty about disease progression
2. Perceptions of Screening Services	• Positive attitudes towards early detection • Trust in faith-based centres • Concerns about confidentiality and stigma
3. Challenges in Implementation	• Affordability of follow-up care • Accessibility and distance to services • Fear of knowing health status
4. Addressing Implementation Challenges	• Role of community health workers • Importance of counselling and education • Need for multi-sectoral support and follow-up

### Awareness and prevalence of diabetes and hypertension

#### Prevalence awareness

Most participants across both focus group discussions (FGDs) and in-depth interviews (IDIs) expressed uncertainty about the prevalence of both type 2 diabetes and hypertension in their communities and faith centres. The majority of participants across the different groups who were interviewed, felt that these were among the most prevalent diseases in the local communities. A congregation member and nurse congregation member elucidated their views on the prevalence in the quotes below:

*I think diabetes is common in this area. It’s a disease that we don’t take very seriously because we don’t know its signs and symptoms. So*,* these days*,* it is common even with the youth. (FGD, Female Congregation Members)*

*The common diseases here are peptic ulcers*,* then hypertension*,* diabetes and cellulitis. These are the major diseases. (IDI-Congregation member nurse-13).*

Faith leaders, elders and youth group leaders agreed with congregation members regarding the prevalence of the two diseases (i.e. type 2 diabetes and hypertension) in the area. They specifically attributed the high prevalence of diabetes to mass under-estimation of its potency. The perception that diabetes was more prevalent among the elderly than the youth was reported by some participants. Faith leaders however held the belief that diabetes affected both older and younger age groups.

*The number one we always hear of is malaria. The others are typhoid*,* stress*,* diabetes among the aged and high blood pressure (hypertension). At times they go and check their BP and say it’s high.* (IDI-Elder-2)

*The common diseases are hypertension*,* diabetes*,* hepatitis B and C*,* malaria*,* typhoid and others.* (IDI-Youth Leader-18)

Some participants perceived that the two diseases, but especially type 2 diabetes was common across all age groups.

*Really*,* when we were young*,* these were the two that were common with the aged*,* specifically those who are fat. They have everything to their disposal and ate whatever they wanted. So*,* they were prone to type 2 diabetes; but now it is like it is common*,* among the youth as well.* (FGD-Male congregation members)

*I can say BP is high because if you observe keenly now a days*,* the stroke is also common*,* and BP is the leading cause of stroke. So that is why I said BP is high in this area. (****FGD-Female congregation members****)*

#### Perceptions and knowledge of type 2 diabetes and hypertension

Diverse views were expressed by participants regarding type 2 diabetes and hypertension and the risks to health. While some described these conditions as very dangerous, others perceived the two diseases as silent epidemic diseases.

*I think diabetes is a slower killer unlike any other disease because when you have it*,* you don’t know the signs and symptoms and before you realise*,* it will kill you. (FGD*,* Female Congregation Members)*

*For hypertension*,* it’s very common and the highest cause of death in this place because most of our people are dying of hypertension.* *(IDI Congregation member Nurse-11)*

*My wife has diabetes and anytime it attacks her it is always deadly.* *(FGD-Male congregation members)*

The majority of participants, including most congregation member nurses, perceived minimal stigma surrounding type 2 diabetes and hypertension. They believed these conditions could affect anyone, particularly the elderly, and noted that people with these diseases were generally treated with sympathy and encouragement rather than discrimination.

*“People do not point fingers at them. They rather pity them and so render help to them and people try to give them words of encouragement.”****(FGD, Female Congregation Members)***.

*“Here*,* it is normal if they see someone with hypertension or with diabetes it is normal. They do not discriminate against them.”(IDI*,* Faith Leader)*.

*“In my community*,* I didn’t see somebody who is having diabetes or BP and people begin to isolate themselves. I think people consider these diseases as normal like the other diseases such as malaria.”(IDI*,*Elder)*.

*“Stigma is not really an issue for diabetes or hypertension. People know that these diseases can affect anyone*,* especially the elderly*,*so there is no reason to treat people differently because of it.”*(*IDI*,*Congregation Member Nurse)*.

However, a few participants, including the congregation member faith nurses, felt that stigma was still present in some communities, leading to social labelling and gossip. Some participants described experiencing others pointing fingers and openly discussing their condition.

*“Well*,* once it spreads*,* they say it. If I am passing and someone knows that I have it*,* that person will tell the other that I have ‘sikili jaweo’ (diabetes). It is just the pointing of fingers.”* *(IDI*,* Congregation Member Nurse)*.

Additionally, misconceptions about diabetes persisted in some communities, where spiritual beliefs influenced how people viewed the disease.

*“Some people believe that if you are diagnosed with diabetes*,* you must have done something wrong spiritually. This belief makes patients feel ashamed to talk about their condition.”* (IDI, congregation member Nurse).

*Because they don’t know the cause of diabetes*,* they think that it is a curse from their gods so as a result*,* they disassociate themselves with them. So*,* they don’t get that companionship that they need.* *(FGD-Male Congregation Members)*

Some participants also shared concerns that diabetes was perceived as contagious, leading to social distancing behaviours.

*“I heard people talking about diabetes*,* that if you have it*,* and if you are with your children*,* you even have to be drinking from your own cup because it is contagious. Therefore*,* people stigmatize you.”* *(FGD, Female Congregation Members)*.

#### Management of diabetes and hypertension: healthcare services and Traditional/Home-Based approaches

Some participants in both the FGDs and IDIs described the measures adopted to manage diabetes and hypertension at the health facilities, within healthcare services. The most reported approach was the prescription of medication. Additionally, participants highlighted blood sugar testing and exercise recommendations as key aspects of diabetes and hypertension management. Less frequently mentioned strategies included dietary advice, physical activity, and adherence to prescribed treatments.

*“I am a BP patient*,* and it is the orthodox medicine that helped me. They gave me some drugs to take and advised me to monitor my BP regularly.”* (FGD-Male Congregation Member).

*“The person would be kept on medication and given guidance on lifestyle changes. If they follow the doctor’s advice and take their medication*,* that is how they would be treated.”* (IDI-Congregation Congregation Member Nurse).

While most participants regarded hospital-based care as the preferred approach, some also described home, and traditional remedies used for managing these conditions. Commonly mentioned practices included herbal preparations and dietary modifications, with specific food items such as garlic, vegetables, bitter kola, dawadawa (african locust bean powder), moringa leaves, smoked fish, fruits, and guinea corn being recommended for individuals with diabetes. Some participants believed traditional treatments were more effective than conventional medicine.

*“For diabetes*,* they have herbs you can boil and take*,* which makes you urinate a lot and clears the disease. We should eat local foods like dawadawa*,* smoked fish*,* and fruits. We should avoid foreign foods.”* (FGD-Female Congregation Member-04).

A few participants suggested that combining traditional and conventional medicine could be more beneficial.

*“When you asked about orthodox and local medicine*,* I would say that using both together could help because both of them actually work.”* (FGD-Male Congregation Member).

However, congregation member nurses strongly discouraged the use of traditional remedies, citing concerns about safety and efficacy. They emphasized that hospital-prescribed treatments were evidence-based and safer for patients.

*“There are herbal concoctions*,* and because we don’t know how safe and effective they are*,* we advise people to go to the hospital for tried and tested medicines.”* (IDI-Faith Leader).

### Perceptions of screening services

#### Views on screening

The majority of participants from both FGDs and IDIs expressed positive views about the screening programme for type 2 diabetes and hypertension at the faith-based centres in the study area. They held that the screening programme was a suitable approach that could help people to know their diabetic and hypertension statuses, which could help them take appropriate measures to improve their health. According to participants, they were willing and ready to support a screening programme of this type.

*To me*,* I think the screening is good. For example*,* let’s say I unknowingly have a disease hidden in me*,* through the test*,* I will get to know it and that can save my life.* *(FGD-Female Congregation Members)*

*It will be good for me to test and know my health status*,* which can help me to put in place some preventive measures.* *(FGD-Male Congregation members)*

*It is possible because there are many people in the church and so when the health personnel laisses with the religious leader*,* they will be able to find a convenient time and place to do it and then announcement will be made*,* and more people will come to participate* *(FGD-Female Congregation Members)*.

Faith leaders, congregation member nurses, and elders in the IDIs also endorsed the screening programme, considering it as a laudable initiative that could help individuals identify illnesses they may be unaware of. They emphasized the significance of early diagnosis and seeking medical care for effective management as demonstrated in the excerpts below:

*It’s a welcome idea; I know the importance of going to hospital to check oneself. So*,* you coming here to do that*,* I think*,* is a very laudable thing.* *(IDI-Elder-02)*

*It is very important because knowing your status is a way of helping you to seek medical care early to cure the condition.* *(IDI-Faith Leader-05)*

*It is very good or even excellent because when that is done it will help everybody to get access to the screening service.* *(IDI-* Congregation member *Nurse-12)*

Some participants reported that because of the cost involved, they were not able to go for screening services at the health facility. Therefore, these individuals felt that the free screening services could assist them, as illustrated in the following quotes:

*In this church*,* we’ll be really happy if they come to do it for us for free because some of us are willing to go for the test but due to the costs we cannot go and do it.* *(FGD-Male Congregation Members)*

*I don’t know how this screening is going to be done*,* but if it’s going to be free*,* it will help the people because some of them cannot afford to pay for the screening at the health facility.* *(IDI-Youth Leader-16)*

While both congregation members and faith leaders were supportive of the screening programme, their perspectives highlighted different priorities. Compared to faith leaders, congregation members focused more on practical and personal concerns—such as convenience, cost, wait times, and reassurance about confidentiality—as central to screening acceptability. Faith leaders, by contrast often framed the success of screening programmes around their role as trusted messengers, suggesting that their endorsement would significantly influence uptake. This distinction suggests that implementation strategies should address both relational trust and logistical accessibility to maximise participation.

#### Screening within faith centres

Varied opinions were expressed by participants when their views were solicited regarding the screening services through faith-based organizations in the area. While some participants were of the view that the programme should be implemented at the community level, others maintained that it should be implemented at both the community level and faith-based centres. Participants who advocated for the programme to be done at the community level claimed that this could help identify many people who had the diabetes and/or hypertension.

*In support of what the other person said*,* it’s good that you do this programme in both the churches and the communities. Not everybody can come to the church*,* so it will be better if it is done at the communities as well.* *(FGD-Female Congregation Members)*

*If you decide to do it in the churches or mosques*,* what about the traditionalists? It means they’ll be left out.* *(IDI-* *Congregation member* *Nurse-15)*

*We don’t know the nature of your work*,* but*,* doing it in both the faith-based centres and the communities will be good because some do not attend church.* *(FGD-Male congregation member)*

Some participants however felt that using the faith centres was a better option to attract many people to patronise the programme because congregation members would trust the screening officers. They added that congregation members were the same as community members and that they did not see anything wrong if the programme was done at faith-based centres:

*If you do it at the faith centres*,* you will get many participants but if you use the community*,* many people won’t come* *(FGD-Female Congregation Members)*.

*If you’re coming to the church*,* then it’s a good thing because the people in the community are the same congregation members.* *(IDI-Women Group Leader-17)*

*I think if you just walk to someone’s home to talk about this*,* they may not trust you but there will be trust if you pass through our mosque because members will think that the people who have come for the screening are genuine* *(FGD-Female Congregation Members).*

### Challenges in implementation

Participants from both FGDs and IDIs, particularly community leaders and faith-based health workers, expressed various concerns and perceived challenges about the screening programme. They emphasized that conducting the test without ensuring access to treatment for those who test positive would not be sufficient. As one female congregation member noted:

*To me*,* the testing is nice*,* but I heard you say that you will do the testing*,* and you cannot provide treatment. If you test the person and the person is having the disease and cannot afford treatment*,* I think there is going to be a problem.* *(FGD-Female congregation members).*

Similarly, participants in the IDIs emphasised the importance of collaborative approaches to address treatment costs:

*Cost of treatment will be a problem*,* but when we collaborate with experts*,* it will be done. We can look for sponsorship from elsewhere that can help.* *(IDI-Male congregation member*,* Youth Leader).*

Some participants expressed that both they and other congregation members would be reluctant to participate in the screening programme if treatment support was not made available to them.

*If it is for you to come and test and not give drugs at the end*,* I will not test because I have no money to be able to afford the drugs.* *(FGD-Female congregation members)*

*There are some people that when you refer them*,* they will not go. They will read the financial implication of it and that fear will not let them want to go.* *(FGD-Female congregation members)*

However, a few participants disagreed with their colleagues or members on the issue, acknowledging the value of the screening in helping individuals know their health status, even in the absence of immediate treatment support.

*We are happy that people are coming to screen and see whether they have it or not. Whether the person gives you drugs or not*,* I think it is still good because it will help you to know your status.* *(FGD-Male Congregation members)*

Other implementation challenges were reported by participants. They identified poor education or information about the programme as a potential hindrance to it’s acceptability.

*One of the greatest obstacles that could affect the success of the screening will be poor education or information*. *(FGD-Male Congregation members)*

Time constraints were also mentioned, with concerns that lengthy screening procedures might discourage participation since they would not have much time to spend going through the programme after religious services.

*Time is also one of the challenges. Normally after mass on Sundays*,* some people are always time conscious and want to run away. So*,* if you want to keep them any longer*,* you may have issues with them*. *(FGD-Female congregation members)*

Maintaining confidentiality of health information during the screening was another important challenge raised by participants. This concern was particularly pronounced among congregation members in FGDs, who worried about being publicly identified or judged within their faith settings.

*The challenge will be the issue of confidentiality. There are people who do not want others to know that they have certain diseases. So even if you go to convince the person to come*,* the person will refuse because of the crowd at the screening centre. (FGD-Female congregation members)*

*Confidentiality will be our concern because I will not be happy if you know my health status and go out there and broadcast it.* *(FGD-Male congregation members).*

Building on these concerns, clear differences emerged in how faith leaders and congregation members discussed issues of stigma and trust. While both groups raised concerns about stigma, congregation members more frequently associated health screenings with fear of being labelled as ‘ill’ or judged by others. In contrast, faith leaders emphasised the importance of building trust and leading by example to encourage participation. As one faith leader explained, “Also, we the leaders should also take part to set precedence for the rest of the congregation to follow. If they are coming to do the screening and sees me myself going through the same screening, they will be encouraged in a way to do it.” *(IDI–Faith Leader).* This distinction highlights the need to address both individual fears and institutional trust dynamics when designing screening interventions.

### Addressing implementation challenges

Despite the challenges identified by participants, several participants, especially healthcare workers and faith leaders in both FGDs and IDIs, highlighted strategies to address these challenges and to improve acceptability of the programme. They emphasized the need for treatment support to encourage many people to come for the screening programme. Others emphasized the importance of thorough education to ensure a better understanding of the screening programme. Maintaining confidentiality of patients’ information could also encourage people to support the screening programme.

*Everybody is almost sick*,* and they will want to know their health status. So*,* if you educate them well*,* no matter which day*,* they will come* ***(FGD-Male Congregation members)***.

*To me the information should be made to reach everybody*,* and we should let them understand why the screening programme.(IDI-Faith Leader-07)*

*I think if they can keep things confidential then it would be good.* *(FGD-Male congregation members)*

On the issue of time, participants recommended that enough health workers should be engaged to assist in the screening to reduce the length of time people would spend at the screening centres. Some participants advised that nurses and other health workers within religious organizations should be engaged to support.

*The issue of time can be overcome if the health workers are many who will do the screening so that people can quickly be attended to. If people get to know that the screening programme is moving faster and they do not have to stay longer to be attended too*,* they will come.* ***(FGD-Female Congregation Members)***

*It is important for you people to talk to the nurses in the church to help in the screening activities and for it to go on fast. (IDI-Elder-2)*

Counselling was also viewed as another important strategy that could be used to encourage many people to undertake the screening for diabetes and hypertension according to opinions shared by some participants.

*I will suggest that the people should be counselled on the importance of screening to know their health status. When you counsel them very well*,* you will remove that fear in them and they will be able to come out to do the screening*. *(FGD-Female congregation members)*

## Discussion

The findings of this study highlight the community’s acceptance and positivity towards screening and education programmes through faith-based centres in the Kasena Nankana Districts of Northern Ghana. The participants’ recognition of the importance of early detection and management of conditions such as type 2 diabetes and hypertension indicate the relevance and necessity of the programme within the community. Moreover, their connection between religion and health highlights the potential for screening initiatives through faith-based centres. The community’s acceptability of faith-based screening aligns with observations from other researchers examining Black African communities, where religious and community practices have been successfully integrated into health interventions [11, 12].

While Theme 1 on awareness and perceptions of Type 2 diabetes and hypertension was not the central focus of this study, it provides important contextual insights that help interpret the acceptability of screening services. Participants’ misconceptions, limited knowledge about risk factors, and uncertainty around disease progression may influence their motivation to engage with screening. Similar findings have been reported in other Ghanaian and African settings, where low disease literacy has been identified as a barrier to early diagnosis and preventive action [28]. Moreover, faith-based organisations have been shown to play a central role in bridging knowledge gaps and delivering culturally relevant health education [10]. These findings reinforce the need to embed tailored educational components within screening initiatives to strengthen understanding and encourage participation.

However, there were notable challenges to the implementation of the screening and community acceptability that need to be addressed for the programme’s success. A key barrier identified was the lack of adequate information and education about the screening programme. To overcome this, a multi-faceted community education strategy should be implemented. Drawing from the successful *Seasonal Malaria Chemoprevention* (SMC) intervention in Northern Ghana, which demonstrated the importance of continuous and intensive health education [15], it is crucial to maintain similar ongoing education efforts tailored to cardiovascular health. Educational campaigns should be community-specific, utilizing local media, faith leaders, and community health workers to enhance understanding and address misconceptions about screening [10, 24].

Another challenge identified in this study was a lack of trust and concerns about confidentiality, similar to findings from other research in Uganda where community members feared health screenings due to associations with other diseases, such as HIV [16]. Building trust within the community is essential for overcoming these barriers. Participants in our study expressed concerns that discussing health status publicly within faith settings could compromise confidentiality, deterring individuals from attending screenings. Ensuring that faith-based health workers are adequately trained and supported, can help mitigate these concerns by reinforcing confidentiality measures and fostering trust between community members and healthcare providers. Ongoing community engagement, coupled with feedback loops, could further bolster trust. However, trust alone is not sufficient to guarantee participation. In addition to these concerns, logistical barriers such as long distances to screening sites further hindered participation, exacerbating fears about knowing one’s health status. These logistical challenges highlight the need for additional measures to ensure accessibility and convenience for the community.

A key challenge reported by participants was the fear of knowing one’s health status, which they perceived as a potential deterrent to participation. Additionally, the long distances to screening sites further compounded these apprehensions. These findings emphasise the need for community-specific strategies, such as targeted education campaigns and community engagement efforts, to bolster the acceptability and utilization of the programme. Drawing parallels from both [13] and [14], it is evident from this study that community trust, coupled with appropriate training and continued support, is pivotal in ensuring the success of community-based health interventions. Pre-screening counselling sessions could help reduce fear, while post-screening support, including health management counselling, could alleviate anxiety following diagnosis. To address logistical barriers such as distance, mobile health units or additional support for community-based outreach initiatives could bring screening services closer to underserved groups.

Participants emphasized the importance of post-screening counselling and support to reduce anxiety and facilitate appropriate health management for individuals identified with Type 2 diabetes and hypertension. Evidence from a faith-based screening and education programmes in rural African American communities highlights the effectiveness of integrating health education, individual counselling, and follow-up support within faith settings to reduce the burden of cardiovascular disease and diabetes [25]. These findings align with broader research demonstrating the benefits of culturally tailored, faith-based interventions in improving chronic disease management, such as the FAITH! App, which successfully promoted cardiovascular health among African American communities [26]. In the Ghanaian context, support for post-screening care could be enhanced through task-shifting strategies and integration into existing primary health structures, such as CHPS. The Task-Shifting Strategy for Hypertension Control (TASSH) has demonstrated success in decentralising follow-up care and empowering nurses to deliver effective NCD management in community settings [27; 28].

Despite the potential benefits of faith-based screening, participants identified financial constraints as a key barrier to accessing necessary treatment. The provision of comprehensive health education and financial support was highlighted as crucial in addressing challenges within the post-screening referral process. In particular, participants recommended that faith organizations play a more active role in supporting individuals with treatment costs, reinforcing the need for multi-sector collaboration in health service delivery [9]. These insights suggest that sustainable models of faith-based health interventions should incorporate structured referral pathways and financial assistance mechanisms, as highlighted in our other paper on this study emphasizing the need for stronger linkages between faith-based screenings and formal healthcare systems [18] Engaging faith organizations and advocating for targeted government funding could help address these financial barriers, ensuring equitable access to care in underserved communities.

Participants expressed mixed opinions regarding the incorporation of educational messages into religious services. While some were open to the idea of using religious platforms to disseminate health information, others were concerned that it might disrupt religious activities, particularly fearing that time constraints during services could compromise the effectiveness of these messages. Allowing faith leaders flexibility in how and when these messages are delivered, and involving faith-based health workers, could address these concerns. Previous studies, such as those conducted in South Africa [12], have shown that integrating health education with religious practices can increase participation and lead to positive health outcomes. Furthermore, faith-based health workers, including congregation member nurses and religious health professionals, in both churches and mosques, can play a pivotal role in disseminating this information during services and thus play a pivotal role in delivering culturally sensitive health education. Their trusted positions within the community allow them to bridge the gap between religious teachings and medical guidance, ensuring that congregation members receive accurate information on the causes, symptoms, health implications, treatment options, and preventive measures related to cardiovascular conditions [10, 11].

Overall, this study highlights the importance of addressing implementation challenges and community acceptability to ensure the success of the cardiovascular screening programme in faith-based centres. By incorporating religious perspectives, engaging faith leaders, and providing post-screening support, the programme can be better tailored to meet the needs and preferences of the community, resulting in improved health outcomes for individuals affected by type 2 diabetes and hypertension. This approach not only fosters trust but also increases the likelihood of sustained engagement in health promotion initiatives within faith communities. These findings also offer broader lessons for other low- and middle-income countries (LMICs), highlighting how community-based screening in religious settings can serve as a culturally anchored and trusted approach to improving awareness, early detection, and management of chronic conditions.

### Policy and practice implications

The findings of this study have several implications for health policy and practice in Ghana. First, policy frameworks must support partnerships between health services and faith-based organisations to embed cardiovascular disease (CVD) screening within trusted community settings. The positive response from both congregation members and faith leaders indicates that religious institutions can act as effective platforms for health promotion, particularly in underserved areas. Second, Ghanaian health systems have initiated efforts to integrate screening and post-screening services for NCDs into the existing CHPS. While this integration is ongoing and more advanced in some regions than others, CHPS provides a viable platform to support decentralised NCD management and follow-up care. Building on these efforts, there is a need to further strengthen and scale up this integration to ensure consistent follow-up care, counselling, and referrals after screening. These primary healthcare units are well-positioned to support decentralised management of NCDs, particularly when supported through task-shifting strategies. The TASSH, for example, has shown that with appropriate training and support, community health nurses can effectively manage hypertension and deliver ongoing care in local settings (27, 28).

Finally, faith-based organisations should be engaged not only as screening sites but also as active partners in delivering health education and post-screening support. Government actors, including district health management teams, should develop mechanisms to involve religious networks in planning, co-delivery, and funding of community-based screening initiatives. This could include training for faith-affiliated health workers, resource allocation to CHPS linked to congregations, and formal referral protocols between faith-based centres and health facilities. This aligns with findings from our earlier paper [18], which highlight the feasibility of implementing collaborative models between faith-based centres and formal healthcare systems to expand screening and linkage to care for chronic conditions in Ghana. Together, these studies form part of a wider programme evaluating faith-based strategies for chronic disease prevention and management in underserved communities.

### Limitations

A key limitation was the potential bias introduced during transcription and translation from local languages (Kasem and Nankani) to English, which may have influenced the consistency or nuance of thematic interpretation. While we took steps to mitigate this—such as involving bilingual research team members to review the recordings and transcripts for accuracy and meaning—there remains the possibility that subtle cultural or linguistic nuances may not have been fully preserved in translation. This is a common challenge in cross-language qualitative research, and future studies may benefit from involving local interpreters throughout the analysis stage to further ensure cultural and contextual fidelity [29]. Another challenge was the limited availability of faith leaders, causing minor delays in scheduling. Future studies should consider logistical support, such as additional community workers, to facilitate timely data collection. There is also the potential for social desirability bias, as interviews and focus groups were conducted within faith-based centres and, in some cases, facilitated by known community members or researchers affiliated with the study. While some members of the research team were external to the local community (including researchers from the UK), others were locally based, which may have influenced how participants responded. However, the use of experienced facilitators, reassurances of confidentiality, and the range of responses expressed, including both supportive and critical views, help to mitigate this risk.

The use of purposive sampling may have introduced bias, as the views might not fully represent the wider population across the districts. Nevertheless, by selecting participants from diverse age groups and religious backgrounds, we aimed to capture varied perspectives. While not statistically generalizable, the findings offer valuable, in-depth insights for understanding community-specific interventions that can inform similar approaches in comparable settings [30].

## Conclusion

The findings from this study demonstrate substantive community acceptance and optimism towards the proposed cardiovascular screening programme in faith-based centres within the Kasena Nankana Districts of Northern Ghana. Participants recognized the importance of early detection and management of conditions such as type 2 diabetes and hypertension. This integration of faith-based centres into healthcare offers a promising pathway to improve health outcomes, particularly in settings where access to traditional healthcare services is limited.

To ensure the programme’s success, it is essential to address key implementation challenges, including the need for continuous community education, building trust, protecting confidentiality, addressing logistical barriers, and overcoming financial constraints. Future research should prioritise piloting and evaluating the proposed screening model to assess feasibility, cost-effectiveness, and health outcomes, such as blood pressure control, treatment adherence, and service uptake—and to inform long-term scalability across faith groups and geographic areas. This includes examining how faith-based approaches can be integrated within Ghana’s CHPS and adapted to address other NCDs. Research should also focus on testing the long-term sustainability of these models and their effectiveness across diverse religious and cultural contexts.

## Supplementary Information

Supplementary Material 1.

Supplementary Material 2.

Supplementary Material 3.

Supplementary Material 4.

## Data Availability

A sample of one de-identified focus group discussion transcript is provided as a supplementary file. To protect participants’ privacy and anonymity, the complete set of transcripts is not available.
