# Supplementary material for: Community acceptability of cardiovascular risk screening in faith centres in the Kassena-Nankana districts of Northern Ghana: a qualitative study
Source: BMC Public Health. 2025 Nov 5;25:3792. doi: 10.1186/s12889-025-24780-z (PMC12587704; doi:10.1186/s12889-025-24780-z)
Supplement: Supplementary file 1 — Supplementary Material 1. [file 12889_2025_24780_MOESM1_ESM.docx]

FGD WITH 19-39 FEMALES AT BASILICA

**I: MODERATOR**

**R: RESPONDENT**

I: Is there a link between religion and health?

R8: I think there is a link between religion and health because in the olden times when religion started, they used to rely on some religious beliefs to cure some diseases and also some people believed in some religious leaders to health them. So that is the link between religion and health.

R7: I also believe that there is a link between religion and health because the traditionalists used to say that there are certain foods that are forbidden to be consumed by some people especially women. For instance; women are not supposed to eat eggs or meat especially chicken so you see that they would not get the required nutrients that are needed for the healthy growth of the mother and the unborn child. So, I think it has a link because if you are not able to get the required nutrients, at the long round, the women can fall sick or develop certain health complications.

R1: I think there is a link between religion and health because mostly when we go to church, they preach and you are happy and that alone heals. You see the cardiovascular diseases for instance, hypertension usually aggravate when the person is annoyed but when you go to the church and your heart is at peace, it makes you comfortable and you easily forgive. That way, your temper goes down automatically and those diseases that are always more pronounced when a person is angry will be controlled.

R8: When we were kids, in the traditional homes they used to say you cannot talk whiles eating and I think there is a link because when you talk whiles you eat, you can be chocked by the food. Also, they told us that it was prohibited to sing whiles bathing and all that was meant to protect your health because when you sing whiles bathing, the soap can enter your mouth. So that is the link.

R3: I think religion and health go together because when we were learning catechism, we were taught that when you are sick, you have to go to treat yourself before you come back to the church. If you are not well, you cannot talk to God. What will you say to God? If there is someone or drugs that can cure your ailment, you have to treat yourself before you can come back to the church. You don’t have to come to the church to do other things which may jeopardize your health.

I: What the key factors that work for and against health and well-being in this church?

R7: I want to talk about the negative effects. At times we believe that some issues are spiritual and the church will advise you to fast and all that and this have a negative impact on the person.

R8: I want to talk on the positive aspect and that is, the health researchers can organize programmes and talk to us about health just as you have come to do. As many people come to the church, the health people can come and educate them on some of the common diseases. That can help the church a lot. We have other organizations coming to the church and asking for permission to advertise their medicines and to do screening. So, if this is done, people will come to participate and it will help them get to know even diseases that they are not aware of. This will enlighten them to know more about their health.

I: What are the key public and private entities that influence health and well-being among people in your area?

R7: Navrongo health research center is one of them. As you are here, you want to screen people for diabetes and hypertension. Not only this, they go to the communities to educate people about diseases and how to practice personal hygiene to overcome such diseases.

R1: The social media also helps a lot especially the radio stations. If there are outbreaks of certain diseases, they announce and educate people about those conditions so that they know what to do to overcome such conditions.

R2: I think the church is one of the organizations that help in health-related matters. When there is an outbreak of any disease or problem, they announce it in the church for others to hear because latterly not all people listen to radio and all that and they will enforce the congregants to strictly observe and obey everything that the health professional will instruct them to do. For example; when covid set in, most of the churches placed notices on the gates saying no mask, no entry. So, that way when you are coming to church, you will be compelled to wear a mask. So, I think they contribute to health and well-being.

R8: The local authorities like the chiefs also help in the prevention of diseases. Sometimes the health facilities rely on them to organize health programmes for them.

R5: social media like WhatsApp, Facebook and the rest also give us information on the outbreak of diseases and how to prevent them.

R6: Community health providers. We used to see them come around to educate people on health issues including vaccines for the different age groups depending on who qualifies to take them. They will normally educate you on the health benefits and encourage you to take it. When covid came in, I saw a lot of people going round and educating people on the vaccine and encouraging them to take it.

I: So, all those groups that you have mentioned, what can you say relatively about their contribution to health and well-being in the community?

R8: Relatively, their contributions have been positive. They have helped a lot of people to open up to the health professionals.

R6: I think relatively, we can say it has been positively influential. Those days a lot of people depended on leaves and other things that they boil to take and those medicines have side effects on the heart and in the end, they burden their organs and situations but with all this media around educating us on what to take and what not to take, why not to take herbs and other things that can complicate our health and things is very beneficial.

I: What are some of the most important ways that religion and religious bodies can do to improve health and well-being of members of our church?

R2: I will like to talk specifically on the religious bodies that they need to organize religious programmes and invite the health professionals to educate their members on the diseases and how to prevent them and also encourage them to go for screening to know if they have those diseases so that if they have them, they can know to how to handle them than to sit and die with it.

R8: I will add that religious bodies should make time during their services to educate their members on some of the causes of some of the diseases since busy schedules would not allow some members to attend seminars unlike the church services that they will definitely make time to attend. If we are educated on the causes and the prevention, it would open up our minds to know those ways of life that predisposes us to those conditions. This can be on monthly bases.

I: In which way can we as church members contribute to health?

R7: I think we should also give information to the congregation. Tell them the importance of checkups and screenings so that we will be able to detect early sickness and seek treatment.

R6: I think the churches should also be responsible to encourage people to always seek health care in time as we mentioned earlier that some of us are at home and saying that our problems are spiritual and others are sleeping in churches to get their healing. So, I think the churches should educate these people to know that whatever that is happening to them, they have to seek health care before prayers can then be done to help.

I: So, we are moving on, what are the most effective programmes that your outfit has undertaking in the community?

R9: The people who have been coming to the communities to do that is the community health nurses and the health researchers. These people normally come to the community to talk to people about health issues but the catholic church for example, I haven’t heard of anything like that.

R8: The catholic church is segmented into outreaches and so if there are health programmes, the health personnel in the church are the people who carry out those activities. In the main parish here, we see people coming to do eye screening and other heart problems. This is what I have seen in the main parish here but I cannot talk to what happens at the outreaches though I know they also use the health workers there to run health programmes.

I: Does the church from time to time move out to undertake programmes related to health specifically on diabetes and hypertension?

R1: Yes, it used to do especially at the outstation that I am. For example, in the outbreak of HIV, the catholic charismatic group used to go into the communities to educate people on it and how to prevent themselves from getting it. I was part of those drama clubs.

R2: There is a programme ongoing that the catholic church has organized for the widows and their main purpose is to help those that have dropped out of school. I don’t know if the first batch has completed but the second batch has also started. They give them vocational training and if you are a woman with kids, they take care of the children. They put them in school and fund all their education. So, I think it is a way of removing that burden of not being able to take care of the kids and even the worries like BP and other heart diseases have been reduced because of this relief.

I: So, which one of the programmes that you mentioned that your church undertook that you are very proud of?

R2: The programme that they organized for the widows. A lot of people were at home but currently they are in school. So, I am happy for that.

I: Now, let’s narrow the conversation to the individuals here. What can you do help religious bodies contribute to health?

R9: You can educate those who do not have any idea on the various health conditions and their effects.

R6: We can do one on one education. I think almost all of us here are teachers and so, in our work places and schools, we can educate people. I have always said that I am not a health worker but I am health conscious. So, most of the diseases that I am aware of, I educate people on them and advise them to seek medical care especially these days that everyone has malaria and virtually every month, somebody is treating malaria. And I have been asking people, have you tested? So, I think if we start this one-on-one education with especially those closed to us, it would spread and go a long way to help.

R8: I think we can also identify serious health conditions in our communities and come out with proposals to seek for support from the church and other organizations to execute them for the benefit of our people. Because in the church, the leaders are in the main parish and we are in the communities and know much about what happens over there. So, when we see things like that, we have to inform the leaders as well as come out with ways of resolving those issues for them to support. They can help in making it public and also look out for sponsors for us to carry out that activity. So, I think we should always get detailed information about what happens in the community levels and get every other person involved because health is something that all must put hands on the deck to see to it that things happen the way we want it.

R7: We can also organize our colleagues and educate them on things happening so that together we can then go round and educate the general public.

R5: For me, I have realized that most people when they are sick, they only go out for drugs. They don’t think that there is the need to check and know what is wrong with them. They will go and buy any drug at all and even those drugs dealers would just come around and explain to them and they buy. This one can lead to problems in your life. So in this kind of situation, I think we can advise the person to rather go for checkup before going in for any drug.

R2: We can also keep prompting people to do the right things like going to do screenings.

R6: As part of it, I think we can help clear some misconceptions about health that our people have in the community. Some people say that when you have certain conditions and you take alcohol, it would help you but we know that alcohol has effects on the heart. Some also believe that when you have stomach pain and you take alcohol, it would go. So, as we learn all these things, we can also help to clear these misconceptions in the minds of our people whiles we encourage them to go to the health facilities to seek medical care.

R7: Also, we should practice what have learnt for them to follow suit. For instance, in the outbreak of malaria, the health workers used to come out to clean for the people to see before they know the importance of it. When they see that what you are doing is helpful, they would also want to do the same to keep the environment clean.

I: Thank you so much. Now we want to talk on awareness, prevention and management of diabetes and hypertension. Firstly, I want to know from you, what are the common diseases that affect people in this community?

R1: The common disease that affects people in my area is malaria. Also, BP is a common health issue with the aged.

R9: Hypertension and diarrhoea.

R3: Diabetes, asthma and stroke.

R8: There is also this ringworm that affects children in this community.

I: How common is diabetes in this part of the country?

R7: It is very common with the aged. Some few of the youth also suffer it but it is a common disease of the aged.

R5: One of the common diseases is anemia.

R1: To talk of diabetes, I think it is now common because it is a disease that we don’t take it very seriously because we don’t know the signs and symptoms of it. I think diabetes is even a slow killer than any other disease because when you have it, you don’t know its signs and symptoms and people don’t also do periodic screening to know our health status. So, nowadays it is common even with the youth.

I: How do people get diabetes?

R2: It comes from the food that we take. Sometimes when some people go to the hospital and they ask them to stop or limit the intake of some foods, they find it difficult to come to terms with and will even be asking, what has happened? This is how it started.

R7: I think diabetes is when the body is not able to digest the glucose we take. When there is too much of carbohydrates in the diet and the body is not able to digest it, it causes diabetes.

R3: I heard that people who like taking sugary things get diabetes.

I: So, what kind of diet causes diabetes?

R2: just like my sister spoke on the carbohydrates, most of the people take too much carbohydrates and it is their habit. When you advise them, they will say they don’t want what you are recommending for them, they prefer to take what they are used to and they even take foods with high levels of cholesterol in it.

R7: Take maize for example, often times we take off the nutritious part of it before grinding it for our TZ. The outer part of this cereal contains fibre that aids in digestion but it is always taken off. We don’t want the local rice but prefer to eat the polished one. All these give us the sickness.

I: How do people get to know that they have diabetes?

R7: Unless you go to do the screening.

R8: Mostly when people have sores that do not heal on attempts to get them healed, then they suspect that it is diabetes. For most of the diabetics, their sores are difficult to heal. So through that, they then go to test and are told that it is diabetes.

I: So, apart from going to screen to know your status or having a sore that does not heal, is there other ways that people use to know if they have diabetes?

R1: Actually, because this sickness used not to be very common among us, we don’t know the signs and symptoms of it. Unless you go to screen and they tell you, you don’t know you have it.

I: Now that we know how people get diabetes, how can it be prevented?

R2: I will suggest that we reduce our intake of foods that are high in cholesterol. We will also need health personnel to advise us on the foods that would give us diabetes so that we reduce our intake of those foods.

R6: I think regular checkups would help. We have already mentioned that diabetes is very common so regular checkup will help us to know when one is at risk of getting it.

R8: We have to reduce the rate of intake of carbohydrates and also do regular exercises. This will reduce our chances of getting diabetes.

I: When people get to know of their diabetes status, what are normally their experiences?

R8: You know, getting a strange thing in your body will not make you normal because everyone wants to be in good shape to go about his/her daily activities. So, the experiences depend on the individual and the counselling that they get before the news of their health status is broken to them. If the counselling is well, the person can take it normal and kick start with the treatment regimens without any issue of frustration setting in but if the counselling is not done well, the person can take it to be the worst thing in his life. With this in mind, frustration and health issues can set in.

R2: They easily get tired when they are working and will not be able to accomplish their targets for the day. They usually end up postponing which they will never get things done and as a result, all the time, they are always angry and when you cross their paths and you are not careful, because of the anger they have accumulated already, they will transfer it to you.

I: What do people in the community think about people with diabetes?

R2: Because they don’t know the cause of diabetes, they think that it is curse from their gods so as a result, they disassociate with them. So, they don’t get that companionship that they need.

R8: What I heard is that, people believe that there is no cure for that disease and so when you get it, you will die with it. So, because of that, when they hear someone has it, the explanation that they will give concerning the disease will make the person loose hope. They always say that with that disease hmmm. They have the believe that with that disease your life is 50/50 because it has no cure. So, they are afraid and distance themselves from you and try to find out if your condition is contagious.

R6: My people call it sugar sickness. Sometimes when you are young, they say it is because of your too much intake of sugar that caused it. So, sometimes because of this misconception coming from the local people, you don’t get the needed support to actually seek further help to know what is wrong with you.

I: So, what do you think about testing people to find out if they have diabetes and hypertension?

R6: For me, it is very good. We have already mentioned that regular checkups would be good for us to know our health status so if there is screening, that is good.

R8: I want to add that before the screening, the necessary education is given to the clients because you can’t just start to test without letting the people know the importance of the screening. So, we can do mass education and let the community health volunteers to organize the community members to come for the screening.

I: How are people tested for diabetes in this community?

R7: They take your weight to be able to tell if you have diabetes or not.

R8: They test urine to know the sugar or protein level in it. I have seen that at the antenatal clinics, this is what they do there.

R2: They also check to know your blood glucose level.

I: Now let us look at how diabetes is managed in the home, health facility and traditionally. How is diabetes treated or managed in the home?

R2: They give them special diet and stop them from foods that will give them more problems.

I: Tell me more about the special diet?

R2: The special diet refers to the food like the groundnut soup, TZ and so on that we eat. So, they will look at your sugar level and advise you on what to stop taking and what to eat.

R1: I also heard that in the local homes, if you are diabetic, they advise you to take more vegetables and you don’t eat starchy foods. When you are preparing banku, you don’t add cassava dough. The perfume rice is starchy so they advise you not to take that. They also advise that women cook TZ using the local millet.

I: Can you name those vegetables that they will normally say you should eat?

R1: They normally advise you to take vegetables like carrots, lettuce, cabbage and so on.

I: Thank you. Now let’s talk about health facility management. How is diabetes managed at the health facility?

R8: They test your blood sugar and advise you on the exercises you should do.

R7: In the health facility, they give you the diabetes drugs.

I: Now let’s look at how diabetes is treated traditionally. Tell me how diabetes is treated traditionally?

R7: They said you can grind garlic and put it in water to be drinking every morning.

I: In the community, are there local healers for diabetes and what do they use to treat or manage diabetes?

R5: I learnt there are herbal medicines that cure it.

I: So, how important is food and or diet in the management and prevention of diabetes and hypertension?

R2: Food is very important. We get the glucose from the food so when we monitor our food well, we can control diabetes.

R8: Food is very important because diabetics are always very weak and when they eat food, they will become strong again.

I: So, in your opinion, are they foods that you will encourage that people eat more and foods that you would discourage people from eating?

R7: I will encourage that they eat more vegetables and foods that contain more roughage to aid in digestion. We should discourage people from eating too much carbohydrates like banku, TZ, and so on.

R2: We should be discouraged from depending more on modernization because in the olden days, it was not so common to get things like that. They could only consume what they farm but now because of modernization, we are all moving with the world. We are throwing away what is good for our health and rather eating what is causing a lot of health issues for us. We should go back to our olden culture. I think that would help us.

I: Now we want to talk about views on the use screening for diabetes and other cardiovascular conditions. screening for diabetes? What do you think about screening for diabetes and hypertension through the church/using faith centres such as Churches and Mosques in this district? Is it something that is feasible?

R2:.

I: If it is possible to screen people for diabetes through the church, then which people should be used for the screening exercise? Should it be faith-based health professionals like the nurses in the church or it should be other health workers coming into the church to screen people?

R8: If the health people see the priest, they will know how to do it. But most of the time, some people do not like other people who know them to hear anything about their health status. So, if you use general practitioners, it will be good.

R5: You are not coming to announce the results to the general public or mention names so I don’t think there will be any problem using nurses in our church.

I: So, assuming we want to use faith-based health professionals for the screening exercise, how do we identify them?

R8: You can identify the health workers in the church through the church leaders. We are in the same church so we know ourselves very well. So, the leaders can assist the health workers to get the health workers in our church by identifying them.

R7: We can use the health members in our church and others outside the church but the problem with using members in the church is that, people are going to say things like; since when have you gone to learn things on health and you are coming to screen us and all that. So, they may not take the results to be authentic. This is because they already have the perception that you are not trained and so how will they then believe the results? So, I think that you can use the health members in our church with other members outside the church.

I: If we want to implement screening exercises in the various churches and mosques, what do you think would be a challenge to this intervention?

R2: Yes, because in everything, there should be something concerning money but now the economy is hard especially the northern part of Ghana. So, I think financially there would be a challenge.

R9: Most of the people may not want to do the test. Even if you say it is compulsory for everyone to take part in the exercise, some will not still do. So, it would be difficult everyone to come for the screening.

I: What about issues of trust, do you think the congregation members will trust the results of the screening exercise?

R7: I think it all depends on those carrying out the exercise. If they are familiar faces, they may not trust the results because they think that they know you and don’t believe you can do that work. But if the faces are unfamiliar and they are health workers, they believe that they are professionals and so would not lie to them and also if they test and there are issues with their results, no one would hear.

R8: Time is also one of the challenges. Normally after mass on Sundays, some people are always time conscious and want to run away. So if you want to keep them any longer, you may have issues with them.

I: So, all these challenges that you have mentioned, what can we do to overcome them?

R8: The issue that has to do with time can be overcome if the health workers are many for the screening exercises so that people can be attended too as fast as possible. If people get to know that the screening exercise is moving faster and they do not have to stay longer to be attended too, they will come.

I: So, on the time, what time do you think will be best for this activity? Should it be immediately after the church service, during the service or some other day after the Sunday?

R8: Immediately after the service would be best if the health workers are readily available because if the members leave the church premises, it would be difficult to get them unless the following Sunday. So, we can do it immediately after mass in smaller groups so that it can be done quickly and the people will go home.

I: So, what concerns will people have after knowing their diabetes status?

R1: Some of them will be worried because some people don’t even want to know their status. If you don’t know something, you don’t bother about it but there are some people who don’t just want to know anything about their health status so that it would disturb them. So, most of them would be worried after they are told that they have diabetes.

R9: They will be worried about how to get the medications and the required diet to live on.

I: So, what can we do collectively to help reduce these fears?

R7: I think if the health people can come around and educate the people on the local foods that are good for diabetes, it would be better than allowing them to buy imported foods that would worsen their situations.

R8: The church and the other bodies should help to absorb some of the cost of the drugs. This will help those who have diabetes to get full treatment. The churches too can also make offerings or collections to raise funds to support the treatment of such diseases.

R6: It is the same thing that I wanted to say. The church can have a fund like that to help people with certain conditions. If there are funds and people come with such conditions, the church can pick money from the fund and see to it that they are given medical attention. You know diabetes is not something that can be treated once and for all. So, if there is fund and the cost of treatment gets tough, the church can assist people to pay for the cost of treatment in order to live long.

I: After screening, we will definitely come back to you with the results, how do you want the results to be shared? Should it be done one on one, or in aggregates?

R8: It should be shared one on one with the individuals and in aggregates with the leaders. If you mention the results to the whole congregation, it would put fear in them.

R6: One on one is fine so that you will get to know your personal status. I will also go in for the aggregates because sometimes it tells what is around us. When they say this percentage of people have this or that, it would prompt us to start checking our lifestyles to know how we can manage our situations.

I: So, what are the factors that would affect the acceptance of the screening exercise?

R2: Yes, because most at times before an exercise is undertaken there is always false information that would be given concerning it so if you don’t think twice, it would be difficult for you to do the exercise. Just like covid 19 when it came, they started saying that the government wanted to give women family planning so that they will never give birth and all those things. So, there were a lot of obstacles that were hindering the exercise but if there is proper education, it would help.

R7: I think fear is one of the factors. There is this fear in people that they don’t want to know their health status at all.

R8: Another thing has to do with issues of confidentiality. People are likely to be worried about what will happen to their results and all that.

I: What suggestions do you have to make this screening exercise more acceptable?

R7: I will suggest that the people should be counselled on the importance of screening to know their health status. When you counsel them very well, you will remove that fear in them and they will be able to come out to do the screening.

R8: Also, on the trust that I spoke about, if there is pre and post counselling, it will take care of the issues regarding trust. Another thing is that the people should be given the chance to do confirmatory tests with other trusted health personnel to be sure of their results.

I: Who are the stakeholders that should be consulted to make this screening exercise a success?

R7: I think the rector of the parish and his priest should be contacted.

I: So, what will be the role of the rector in this exercise?

R7: The rector gives the go ahead to the other people and they will in turn organize people to come for the screening exercise.

I: So, apart from the rector who is the head of the priest and the priest, who else should be contacted to make this project work?

R8: I think we those who have been shortlisted for this interview to get the first-hand information about the project should be contacted. We can assist to educate the people based on the information we have had in the previous interview. As we have learned here, the education has started like that and it should spread to our families, friends and church members.

I: Now, we want talk about counselling. Should there be counselling as part of the screening services?

R6: Yes, because in the process of counselling, it helps to reduce fear and whatever perceptions people might have about the screening that is going to be done and that will help people to accept and go in for it.

I: Thank you so much. So, if counselling is important, how should it be done?

R7: It should be done one on one because people don’t want their personal issues to be made known to other people. It would be confidential when it is done one on one.

I: When should the pre-counseling be done?

R7: I think it should be done after you have been screened because this is where you tell especially those who would have it what to do to live long with the condition.

R1: There should be group counseling before the screening and individual counseling after the screening.

I: Which place do you think would be suitable for the counseling?

R1: The church can be used for the counseling because in the church, we sometimes have programmes and you can talk to people before the programme starts.

I: How do you want the counseling to be done?

R1: I think it should be done using the various societies in the church because in the church, everyone belongs to a society.

I: After the screening exercise, if people are found to have diabetes or hypertension, they will be referred to health professionals. Do you think if people are referred, they will like to go?

R2: They will like to go because it concerns their health and when you are not healthy, nothing goes on in life. Health is life.

R6: To some extent, there are some people that when you refer them, they will not go. They will read the financial implication of it and that fear will not let them want to go. They will ask questions like; if I go and they say buy this and I don’t even have the money, how do I buy those drugs and all that. So, people will not like to go. It also depends on the setting. At some areas you may get people who will go but others won’t go.

I: So, apart the fear of financial challenge that would prevent people from going, what else? What can be done to motivate people to go?

R2: Medications should either be provided for free or there should be reduction in the cost of the medications so that those who need to get them can buy.

R6: The people should be assured that when they come, they will get assistance in the purchase of drugs and all that. There was a time they did eye screening here and part of the things like eye drops, eye glasses were given for free and when they refer to the health facility, there were things that they will still take care off and they were made to know of certain things. So, when they know when they come to the hospital, part of their cost would be taking care off, they will accept to go for the referrals.

I: Now let’s talk about the educational messages that would be designed to create awareness about diabetes, hypertension in the faith centers. How do you think we can best do to create awareness about diabetes and hypertension for people to come out willingly to do screening services?

R6: I think fliers and posters can be designed where you put the causes, effects of it in picture forms. When people see it, they will know that when I get it, this is what happens to me. That can be a motivation to them to avail themselves for the screening exercise and for further health care.

R2: We can dramatize it.

R7: We can also do announcement in the radio stations.

R6: We can deploy health professionals to the OPD to talk to all persons attending health care on diabetes and hypertension before they go to treat whatever condition that has brought them to the health facility. This will also help.

I: Which people should we use to give out the educational messages? Take for instance this church, should the educational messages be given out by the priests, nurses in the church or any health professional?

R9: For me, I will say that it should pass through the priests so that they would in turn involve the nurses and all the other people whose services would be needed.

R3: I think the health workers should be tasked to do the education. They should go to the radio stations and educate the general public on diabetes and hypertension.

I: Which place should the educational messages be given and at what time of the day? If we want to give the educational message in the church, what part of the service should this message be given?

R9: I think it should be given out after the mass. It can also be given during the time of announcement in the church.

I: We will definitely come to do the screening in the church so we want to know from you, what resources are available in the church that this project can use to help in the execution of this work? Are there things that this church can offer to help?

R1: Yes. Human beings, chairs, space.

I: What suggestions do you have to help us do this work well so that people would willingly come out to participate in the screening exercise and at the end of the day, trust the results that will be given to them?

R7: I think educating them on the importance of taking the screening exercise will be very beneficial to them. As we have been enlightened on diabetes and hypertension, if we also go out to tell people on the need to screen regularly to know their status, it will also help a lot.

R6: I also think the team can solicit for funds that would help enable them make some of the processes involved in this exercise free of charge. Mostly when you say free screening, you will see people coming but when you say pay this or that amount, then people begin to drag their feet. So, if there is a way the team can come in financially to support people, it would be helpful.

I: So, apart from educating and looking for money to support the needy in getting some of the services for free, what else?

R2: I think time is one of the factors that has to be looked at. We should look for the time that would be convenient to the majority of the people. A day like market days, would not be good for most people because they go to the market and when you want to delay them, they will have issues with you.

R6: All of us live in the community so I think when we get back, we should start with the one on one education with our members on the causes and effects so that when the date is set for the screening exercise, we can then remind them to come out for it.

I: Thank you so much for your time. God bless you all.
