# Supplementary material for: Community acceptability of cardiovascular risk screening in faith centres in the Kassena-Nankana districts of Northern Ghana: a qualitative study
Source: BMC Public Health. 2025 Nov 5;25:3792. doi: 10.1186/s12889-025-24780-z (PMC12587704; doi:10.1186/s12889-025-24780-z)
Supplement: Supplementary file 4 — Supplementary Material 4. [file 12889_2025_24780_MOESM4_ESM.docx]

**GUIDE FOR CONGREGATION MEMBERS**

**Study title:** The Scalability of a Faith Centre based model to improve the uptake of screening services for diabetes in rural Kassena-Nankana Districts of Northern Ghana

Formal GREETING!!! We are working with the Navrongo Health Research Centre, and we are pleased to have all of you here today to discuss issues related to diabetes and other cardiovascular disease. The main aim of this study is to facilitate screening activities for diabetes and cardiovascular risk factors through faith centres (Churches and Mosques) in the Kasenan-Nankana Districts in the Upper East Region of Northern Ghana. These screening services are either lacking or limited in Ghana and the Ghanian population have poorer self-management and medical adherence. Therefore, it is imperative to implement screening services and increase diabetes awareness as well as identification through faith centres. The screening exercise will help detect Diabetic patients and advise them to see their health professionals for appropriate management.

We would like to ask for your consent to use the digital tape recorder to record the discussion. This will enable us capture all the issues that we will discuss here and then write it out when we get back to the office.

***INSTRUCTIONS***

*1. After obtaining consent from participants and switching on the recorder, try to make the interview as natural as possible to an informal conversation. It is very important that study participants feel comfortable with you.*

*2. You can change the order of the questions as they appear in the guide the way that you and the interviewee will be comfortable.*

*3. Try to ask all questions and also probe for clarification of unclear statements made by the participants during the conversation.*

**Background Information**

| **Participant ID** | **Age** | **Marital Status**   1. Married 2. Single 3. Divorced 4. Separated 5. Never married | **Religion**   1. Muslim 2. Christian 3. Traditionalist 4. Other | **Level of Education**   1. Never been to school 2. Primary 3. Secondary/Technical 4. Tertiary/Professional | **Ethnicity** | **Occupation**   1. Unemployed 2. Civil /Public Servant 3. Farmer/artisan 4. Trader 5. Other |
| --- | --- | --- | --- | --- | --- | --- |
| 1. |  |  |  |  |  |  |
| 2. |  |  |  |  |  |  |
| 3. |  |  |  |  |  |  |
| 4. |  |  |  |  |  |  |
| 5. |  |  |  |  |  |  |
| 6. |  |  |  |  |  |  |
| 7. |  |  |  |  |  |  |
| 8. |  |  |  |  |  |  |
| 9. |  |  |  |  |  |  |
| 10. |  |  |  |  |  |  |
| 11. |  |  |  |  |  |  |
| 12. |  |  |  |  |  |  |
| 13. |  |  |  |  |  |  |
| 14. |  |  |  |  |  |  |
| 15 |  |  |  |  |  |  |

**Awareness and perceptions of diabetes, prevention and management**

1. What are the common diseases that affect people in this community/district? If not mentioned, P**robe for:**
   1. Diabetes, hypertension and other cardiovascular diseases
   2. How common diabetes is in the area
2. How do people get that disease? How do people know they have that disease?
3. How can the disease be prevented?
4. Do you know people in the community/congregation that have diabetes? Tell me about their experiences.
5. What do community members think about people who have diabetes in this area?
6. What do you think about testing people to find out if they have diabetes and hypertension?
7. How are people diagnosed with diabetes in this community/area?
8. How is diabetes managed or treated in this community? **Probe for**
9. Home management
10. Health facility management
11. Traditional management
12. How important is food/good diet in the prevention and or management of diabetes and hypertension?
13. In your opinion, are there foods one should be encouraged to eat more or avoid when it comes to diabetes hypertension prevention and or management?

**Views on screening for diabetes and other cardiovascular risks**

1. What do you think about us screening for diabetes and hypertension through faith-based centers (Churches and Mosques) in this district?
2. Which people do you think could be used for the screening exercise? **Probe for use of**:
   1. Faith-based centres health professionals
   2. How we could identify them
3. What do you think will be the challenges in implementing this exercise? P**robe** **for**
   1. Trust of congregation members on the screening exercise
   2. Suggestions to address implementation challenges
4. What concerns do you think people will have knowing their diabetes status? P**robe for**
   1. Ways community members, religious leaders and research team can do to reduce those fears.
5. How would you want the information on the diabetes status of people to be shared with them? **Probe for**
   1. One-on-one sharing of results
   2. Groups or aggregate sharing of results
6. What factors do you think are likely to affect acceptance of the screening exercise?
7. What suggestions would you recommend to improve acceptability of the exercise? **PROBE** for suggestion in relation to
   1. Religious leaders
   2. Congregation members
8. Who are the stakeholders that need to be consulted to make this exercise possible? **PROBE** on the role each of them will play.

**Views on counselling procedures**

1. How necessary is it for participants to be offered counselling services as part of the screening or testing exercise?
2. What do you think is appropriate way for the counselling to be done? **PROBE** for one-on-one or aggregate counselling.
3. Where do you think is suitable place for the counselling to be done? **PROBE** for preferred people to be used for the counselling.
4. What other suggestions do you have to make for the counselling to be more effective and convenience to people?

**Acceptance of advice/referral for medical care**

***If a participant is found to have diabetes and other cardiovascular risk factors, we will advise the person to see his/her medical professional for management. We will also educate people on the disease to create awareness and the need for them to take part in the screening exercise the research team is going to implement using faith centres.***

1. What do you think about this process of referring or advising people to see their medical professionals if they are found to have the risk factors?
2. Will people agree to go and see their health professionals for care? Why? *P****robe for motivation to go for care.***
3. What could be the challenges if people are asked to see their health professionals for medical care?

**Educational messages**

***We will design education messages to create awareness about diabetes and the screening exercise at faith centres.***

1. How do you think we can best do this activity to create awareness about the disease and for people to willingly come for the screening exercise?
2. What procedure (s) do you think could be used to educate people on diabetes and other cardiovascular conditions? **Probe for**
   1. The kind of messages or information to give to people about the disease
   2. How these messages should be packaged and delivered
   3. Appropriate place to be used to educate people (probe for possibility of doing this during service/prayers)
   4. Appropriate people to deliver the educational messages (***probe for possibility of using religious leaders such as Pastors/Rev. Fathers, etc***.)
3. What resources are available at faith-based centres to help the study team undertake the screening activities?
4. Based on the discussions we have had with you, what other suggestions do you have for the study team to make the exercise more successful and acceptable to congregation members?
5. Is there anything else you will like to tell us concerning this discussion?

**Thank you very much for your time!!!**
